# The Effect of Smoking on Mineral and Protein Compositionof Saliva

**Published:** 2015-07

**Authors:** Ali Fattahi Bafghi, Ali Goljanian Tabrizi, Peyman Bakhshayi

**Affiliations:** 1 *Department of Otorhinolaryngology, Shahid Beheshti University of Medical Sciences, Tehran, Iran.*

**Keywords:** Saliva, Smokers, Total Protein, Ca, Na, Mg, K, Pb

## Abstract

**Introduction:**

To assess the salivary composition of proteins and minerals in smokers compared with non-smokers.

**Materials and Methods:**

In this study we compared the total protein and Ca, Na, K, Mg, Pb of whole saliva in two groups of men (28 smokers and 31nonsmokers) aged between 29-41years.

**Results:**

Fifty-nine participants were evaluated. The mean age was 33.14±5.32 years among smokers and 32.15±5.12 years among non-smokers (P>0.05). The mean concentration of total protein, Ca, Pb, and Zn of whole saliva in smokers was lower than that in non-smokers, but the difference was not statistically significant (P>0.05). The mean concentration of Na, K, Mg in whole saliva was not significantly different between smokers and non-smokers (P>0.05).

**Conclusion:**

We specified that smoking reduced the value of total protein, Ca and Pb of saliva, however it did not have an impact on Na, K, and Mg of saliva.

## Introduction

Saliva is complemented with several components that create from intact or destroyed mucosal and immune cells, from intact or destroyed oral microorganisms and from blood serum, that result in a complex mixture of a variety of molecules (1). Saliva has several important roles and affects acquired pellicle formation on tooth surfaces, crystal growth homeostasis, bacterial adhesion and plaque formation (2-4). Moreover saliva has lubricating effect that maintains mucosal integrity of the oral and upper gastrointestinal mucosal surfaces, additionally it impacts on physico-chemical defense, antimicrobial defense, and wound healing. To achieve these important tasks various saliva components including proteins, carbohydrates, lipids, and ions interact under fine regulation (5-7). These complex balanced functions may disturb by local or systemic disorders (8). Cigarette smoking is related to several disorders and impacts on various processes, factors, and mechanisms. Electrolyte changes are one set of alterations occurring in response to cigarette smoking. Change in electrolytes occurs at systemic ,cellular and molecular level. Saliva electrolyte changes in smoking have been studied worldwide and showed non-significant difference for saliva sodium, potassium and calcium concentrations in smokers and nonsmokers (9-11).

On the other hand the evaluation of smoking effect on salivary composition showed decreased levels of antibodies and antioxidants in smokers (12,13). Moreover some studies have indicated elevated calcium concentration and calcium phosphate ratio in smokers’ whit greater plaque and calculus formation (14). Additionally another study by Zuabi **et al.** showed elevated concentrations of salivary electrolytes and proteins in patients with established periodontitis,furthermore they specified that smokers show greater disease level and reduced sodium, calcium and magnesium concentrations (15). Previous studies (9-14) have prepared conflicted results about the smoking effect on salivary compositions ,therefore, to address this concern and to shed light on salivary composition in smokers we steered this controlled survey to compare the salivary proteins and minerals in smokers with nonsmokers.

## Materials and Methods

In this case control study, 59 workers (31 non-smokers and 28 smokers) aged 29–41 years, with a daily consumption of eight cigarettes for at least 2 years were enrolled. Participants were excluded if they had a history of cardiovascular, endocrine, gastrointestinal, oral, or respiratory disease or alcohol consumption. Moreover patients that consumed anti hypertension, anti-psychotic and anticholinergic drugs were excluded from the study. The study was approved by the Ethics Committee of Shahid Beheshti University of Medical Sciences. In addition, the study protocol was explained to all participants and informed written consent was provided in all cases. 

**Saliva Collection**

Stimulated whole saliva (using citric acid 2%) was collected 1 hour after breakfast from all participants. The smoking group was asked not to smoke for 1 hour prior to the collection. 

The participants were seated and asked to swallow saliva and then stay motionless while the saliva was collected by suction over 2 minutes into a sterile plastic vial. Saliva samples were immediatel ycentrifuged (1000 g, 10 minutes) at 4 °C to remove cell debris. The resulting supernatants were immediately deep-frozen at –80°C and stored for later analysis.

To evaluate the total protein in the saliva, we used five solutions as follow:

A: CuSO4, 5H2O 1%. B: NaK+Tartrate 2%. 

C: Na2CO3, 20 g + NaOH 4g.

D: Standard dilution, 200 mg protein/L.

E: Folin and Ciocalteu reagent.

Next, 20–200 μg of standard solution was added to 10 test tubes and supplemented to 1 ml using distilled water. Moreover, in one control test tube, 1 ml of distilled water and solutions of A, B, C, and E were added. Then, a mixed solution of A solution (1 ml), B solution (1 ml), and C solution (100 ml) was added to each test tube (5 ml) and left for 10 minutes. Next, Folin and Ciocalteu reagent (0.5 ml) was added to each test tube, and the tube was shaken and left for 30 minutes. Then, using a spectrophotometer, the resulting blue color was evaluated at 580 nm and saved as a standard figure. Finally, the total protein in the collected saliva was evaluated using the same technique.

For the measurement of Na, K, Ca, Mg, Zn, and Pb, we prepared standard and Stoke solutions and then used atomic absorption (680, Japan, background correction (BGC) mode) for evaluation.

***Stoke Solution***

To obtain Stoke solution (1 mg/ml), NaCl (2.54 g), KCl (1.97 g), Ca2CO3 (0.24 g), Mg (1 g in 60 ml HCl), Pb(NO3)2 (1.59 g), and Zn in HCl (30g) was diluted in deionized water (1L). Then, to obtain standard solution, 1mg of Stoke solution was diluted in deionized water and several concentrations were obtained as follows: Na: 0.5, 0.75, 1.0, 1.5, and 2 μg/ml, K: 0.5, 1, 2, and 4 μg/ml, Ca: 1, 2, 3, 4, and 6 μg/ml, Zn: 0.04, 0.08, and 0.16, Mg: 0.4, 0.2, and 0.05 μg/ml, and Pb (NO3) 2:0.075, 0.15, 0.3, 0.6, and 1.2 μg/ml.

***Statistical Analysis***

Statistical analysis was performed using SPSS 20 software for Windows. All values were reported as mean±SD. The statistical significance of differences in salivary total protein and ion levels between smoking and non-smoking men was estimated by t-test. A P-value less than 0.05 was considered statistically significant.

## Results

Totally we evaluated 59 participants.The mean age was 33.14±5.32 in smokers and 32.15±5.12 in non-smokers and difference between two groups was not significant (P>0.05). The mean total protein, calcium and Pb2+of whole saliva in smokers was lower than nonsmokers but the difference was not significant (P>0.05).The mean of Na, K, Mg of whole saliva did not show significant difference between smokers and nonsmokers (P>0.05). 

However, it is noteworthy that zinc concentration of smoker was lower than that nonsmoker, but the difference between two groups was not significant (P>0.05) (Table.1).

**Table 1:** Mean and standard deviation of salivary parameters in smokers and non-smokers

**Group**	**Smokers** **(N=28)**	**Non-Smokers (N=31)**	**P**
Salivary parameters	Mean±SD		
Total protein (mg/ml)	2.85±0.15	3.3±0.12	NS
Ca2+ (mEq/L)	2.46±0.14	3.17±0.18	NS
K+ (mEq/L)	25.64±0.9	25.14±0.56	NS
Mg2+ (mEq/L)	0.443±0.046	0.453±0.035	NS
Na+ (mEq/L)	13.21±0.31	13.22±0.21	NS
Zn2+ (mEq/L)	0.036±0.04	0.046±0.004	NS
Pb2+ (mEq/L)	0.034±0.001	0.027±0.001	NS

## Discussion

Saliva has multiple potential effects on the oral cavity, such as lubrication, anti-bacterial activity, buffering, pH regulation, and protection of the teeth (16). Saliva plays a major role in plaque initiation, maturation and metabolism; moreover, salivary flow and composition impacts on calculus formation, periodontal disease and caries (17). Ca and P are the major inorganic components of plaque, although trace amounts of other minerals such as Na and K are also present (17). 

Cigarette smoke comprises a combination of chemicals containing over 4,000 components including nicotine, ammonia, acrolein, phenols, acetaldehyde, benzopyrene, nitrogen oxides, carbon monoxide, polonium, radium, and thorium. In addition, cigarette smoke contains free radicals that can cause cellular damage (13). In the present survey we observed biochemical changes occurring in the saliva of smokers that can lead to oral and dental tissue injury (17). We showed that the mean concentration of total protein, Ca, Zn, and Pb2+ in the whole saliva of smokers was lower than that in non-smokers. Consistent with our study, in 2012 Abhay et al. showed that the concentrations of total protein, Ca, Mg, and P were reduced in the whole saliva in smokers (14). Moreover, Zuabi et al. found that smokers exhibited a distinct salivary composition, characterized by significantly lower levels of Ca, Mg, and Na compared with non-smokers (15). However, in contrast to Abhay and Zuabi findings the mean of Na, K, Mg of whole saliva in our survey did not show significant difference between smokers and nonsmokers. In an uncontrolled study conducted in smokers by Nakonieczna-Rudnicka et al., non-stimulated saliva was used as a material for biochemical examinations. Selected saliva components, including protein concentration, Ca2+ concentration and pH value both in male and female smokers were evaluated. The authors observed that neither Ca2+, protein concentration, nor saliva pH correlated with sex or cigarette smoking (18). 

The differences between our study and these studies could be attributed to the different techniques that were employed in terms of patient selection, saliva collection,

and biochemical analysis. Saliva collection is a rapid, painless, non-invasive, and economical technique, and yields findings that are reproducible. For these reasons, saliva is increasingly being studied. Furthermore, as described previously, several factors are known to impact the levels of protein and minerals in the saliva. Sevón et al. reported that flow rate has an important role on the level of components in saliva, and showed that the concentration of Na correlated positively with the salivary flow rate, while phosphate, K, Mg, and protein correlated negatively. Moreover, they indicated that Ca was the only electrolyte which had no association with flow rate. 

Additionally Sevón et al. showed that age had no effect on Mg, Na, K or proteins in the saliva (19). Finally, several studies reported that race, age, sex, hormones and pregnancy significantly impacted the saliva protein level (20-24).

The main limitations of this review are that we did not evaluate the impact of gender, medication or underlying disease on the components of saliva. Previous studies in this field have indicated that the principal difficulties in salivary research among an adult population are the great inter-individual variation, the increasing numbers of subjects using medications, and the presence of concomitant diseases affecting salivary flow and composition (14-24).

## Conclusion

We found out that smoking reduced the value of total protein, Ca and Pb of saliva, moreover smoking slightly changed the value of zinc however these changes were not significant.Smoking did not have an impact on Na, K and Mg of saliva.
